# Contribution des avortements et des grossesses extra-utérines dans la mortalité maternelle dans trois hôpitaux universitaires de Yaoundé

**DOI:** 10.11604/pamj.2017.27.248.12942

**Published:** 2017-08-03

**Authors:** Danielle Victoire Tiako Kamga, Philip Njotang Nana, Florent Ymele Fouelifack, Jeanne Hortence Fouedjio

**Affiliations:** 1 Centre Médical de la Garde Présidentielle de Yaoundé, Cameroun; 2 Hôpital Central de Yaoundé, Cameroun

**Keywords:** Mortalité maternelle, contribution, avortement, grossesse extra-utérine, Yaoundé, Cameroun, Maternal mortality, contribution, abortions, ectopic pregnancy, Yaoundé, Cameroon

## Abstract

**Introduction:**

L'organisation mondiale de santé (OMS) estime que chaque année dans le monde 585 000 femmes meurent de complications liés à la grossesse, à l'accouchement, aux suites de couche et à l'avortement (ce dernier contribuant pour 13% des décès maternels). La GEU est responsable de 10% de mortalité maternelle au premier trimestre de la grossesse. Le taux de mortalité maternelle reste élevé au Cameroun, estimé à 782 pour 100 000 naissances vivantes selon EDS-MICS 2011. La contribution de ces deux entités dans la mortalité maternelle étant peu documentée dans notre pays, nous avons entrepris de réaliser cette étude avec pour objectif d'évaluer la contribution des avortements et des GEU dans la mortalité maternelle au Cameroun.

**Méthodes:**

il s'agissait d'une étude rétrospective et analytique. Nous avons colligé tous les dossiers des patientes enceintes et décédées avant la 28^ème^ semaine de grossesse, dans trois hôpitaux universitaires: Hôpital Central de Yaoundé (HCY), Hôpital Gynéco-Obstétrique et Pédiatrique de Yaoundé (HGOPY), Centre Hospitalier et Universitaire (CHU), sur la période allant du 1^er^ juin 2011 au 31 mai 2016, soit sur cinq ans. Les données étaient compilées sur une fiche technique préétablie et testée, saisies en utilisant le logiciel CS pro 6.2 et analysées par le logiciel SPSS 20. Les tests statistiques de comparaison utilisés étaient le Khi 2 et le test de Fischer en fonction des effectifs. Le seuil de significativité était retenu pour P < 005.

**Résultats:**

Tous avons enregistré 524 décès maternels pour 31116 naissances vivantes, soit un taux de mortalité maternelle (TMM) de 1538.9/100 000 naissances vivantes. Sur les 524 décès maternels, 414 dossiers étaient exploitables, parmi lesquels, 100 (soit 24.2%) concernaient les avortements et 24 (soit 5.8%) concernaient les grossesses extra-utérines, ces 2 entités contribuaient ainsi pour 30% de décès maternels (124 dossiers sur 414). L'analyse des 124 dossiers montre que l'âge moyen était de 27.58 +/-6 ans avec des extrêmes de 18 et 48. La tranche d'âge de 20 à 24 ans était la plus représentée (33.1%), suivie de celle de 25 à 29 ans (24.19%). Les célibataires constituaient 75%, les ménagères 36.7 %, de niveau d'instruction secondaire dans 62.5% et multigestes constituaient 36.1% de notre échantillon. Aucune consultation prénatale n'avait été initiée dans 73.4% et seulement 2.4% en avaient fait au moins 4. Les complications ayant conduit au décès étaient dominées par les hémorragies et les infections.

**Conclusion:**

Les avortements et grossesse extra-utérines restent des causes majeures de la mortalité maternelle dans notre pays. Nous recommandons un renforcement de la planification familiale pour limiter les grossesses non désirées et une prise en charge par assistance socio-économique des patientes à risques.

## Abstract (en)

**Introduction:**

The World Health Organization (WHO) estimates that 585.000 women die every year in the world as a result of complications related to pregnancy, delivery, postpartum period and abortion (the latter contributing to 13% of maternal deaths). Ectopic pregnancies are responsible for 10% of maternal mortality in the first quarter of pregnancy. Maternal mortality rate is high in Cameroon, estimated at 782 per 100.000 live births according to EDS-MICS 2011. AS the role of these two conditions in maternal mortality is little documented in our country, we conducted this study to assess the role of abortions and ectopic pregnancies in maternal mortality rate in Cameroon.

**Methods:**

We conducted a retrospective and analytic study. We collected data from all the medical records of pregnant patients and pregnant patients died before the 28^th^week of pregnancy at three university hospitals: Central Hospital of Yaoundé (HCY), Yaoundé Gynaecology, Obstetrics and Pediatrics Hospital (HGOPY), University Hospital (CHU), over a period of five years, from 1 June 2011 to 31 May 2016. Data were recorded on a pre-established and tested technical sheet, collected using CS Pro 6.2 software and analyzed using SPSS software 20. The statistical tests for comparison used were Khi 2 and Fischer test according to the effective sample. The threshold significance level was set at p <0.05.

**Results:**

We recorded 524 maternal deaths per 31116 live births, reflecting a maternal mortality rate (MMR) of 1538,9/100 000 live births. Out of 524 maternal deaths, 414 medical records were workable, including 100 (24.2%)abortions and 24 (5.8%) ectopic pregnancies. These 2 conditions contributed together to 30% of maternal deaths (124 medical records out of 414). The analysis of 124 medical records showed that the average age was 27.58 +/- 6 years, ranging from 18 to 48 years. The age group 20-24 years was the most represented (33.1%), followed by that 25-29 years (24.19%). The singles constituted 75%, housewives 36.7%, with level of secondary education in 62.5% and multigestes constituted 36.1% of our sample. 73.4% of patients didn't undergo any prenatal consultation and only 2.4% had undergone at least 4 consultations. Complications resulting in deaths were dominated by hemorrhage and infections.

**Conclusion:**

Abortions and ectopic pregnancies are the major causes of maternal mortality in our country. We recommend strengthening of family planning to limit unwanted pregnancies and socio-economic support for patients at risk.

## Introduction

La grossesse est un état normal auquel aspirent la plupart des femmes à un moment de leur existence. Cependant ce processus normal et créateur de vie comporte un risque de séquelles et/ou de décès. L'organisation mondiale de santé (OMS) estime que 585 000 femmes meurent chaque année dans le monde, de complications liées à la grossesse, à l'accouchement, aux suites de couche et à l'avortement [1], parmi lesquels 99% se produisent dans les pays en voie de développement, contre 1% dans les pays à revenu élevé [2]. Le taux de mortalité maternelle reste élevé au Cameroun car estimé à 782 pour 100 000 naissances vivantes [3]. L'OMS estime que les complications des avortements sont responsables de 13% des décès maternels [4, 5]. En Afrique, 300000 décès maternels sont enregistrés par an, dont 30 à 40% sont dus aux avortements provoqués [6]. Les grossesses extra-utérines constituent une cause importante de mortalité maternelle au premier trimestre de la grossesse [6]. En dehors des études menées à l'Hôpital Central de Yaoundé (HCY) par Léké et coll [7, 8], peu de données sont disponibles sur la contribution des avortements et des grossesses extra-utérines dans la mortalité maternelle dans notre milieu. La connaissance de la contribution de ces 2 entités dans la mortalité maternelle permettra de proposer et de planifier des mesures préventives et curatives efficaces afin de contribuer à l'atteinte du 3^e^ objectif de développement durable qui est de «permettre à tous de vivre en bonne santé et de promouvoir le bien-être de tous et à tout âge» d'ici à 2030 [9]. Notre objectif général était de rapporter la contribution des avortements et des grossesses extra-utérines (GEU) dans la mortalité maternelle, notamment déterminer la prévalence des décès maternels par avortements et par grossesses extra-utérines, de décrire les caractéristiques sociodémographiques et de rapporter les complications responsables du décès de ces femmes.

## Méthodes

Il s'agissait d'une étude rétrospective et analytique sur cinq ans (du 1^er^ juin 2011 au 31 mai 2016 inclus), menée dans trois hôpitaux de la ville de Yaoundé: Hôpital central de Yaoundé (HCY), Centre Hospitalier et Universitaire (CHU), Hôpital Gynécologique Obstétrique et pédiatrique de Yaoundé (HGOPY). La population d'étude était constituée des dossiers de femmes décédées dans les 03 hôpitaux pendant la période de l'étude. Etaient inclus les dossiers des femmes enceintes décédées de suites de complications liées aux avortements et aux GEU avant l'âge de 28 semaines. Etaient exclus les dossiers des femmes enceintes décédées de toute autre cause non liée à la grossesse, les dossiers dont les informations étaient très incomplètes pour remplir la fiche technique et les dossiers non retrouvés. L'échantillonnage était consécutif et exhaustif. Les registres de mortalité maternelle nous permettaient d'identifier les noms des patientes décédées des suites d'avortement et/ou de GEU. Leurs dossiers étaient par la suite recherchés au service des archives pour avoir le maximum d'informations. Tous les renseignements étaient collectés et reportés sur des fiches techniques. Les variables étudiées étaient regroupées en caractéristiques sociodémographiques, (âge, statut matrimonial, profession, niveau d'instruction), profil obstétrical (parité, nombre de consultations prénatales (CPN), âge gestationnel (AG) en semaines), antécédents (contraceptive, sérologie VIH), les complications à l'admission (métrorragies, douleur pelvienne, choc hémorragique, l'anémie, l'hypotension artérielle, le choc infectieux, les intoxications médicamenteuses), le type d'avortement (spontané ou provoqué), les résultats de la prise en charge des complications liées à l'avortement (médicale, l'aspiration Manuelle Intra Utérine (AMIU), la dilation/curetage, Le curage, laparotomie d'urgence) et les résultats de la prise en charge des complications liées à la grossesse extra utérine (médicale, laparotomie d'urgence, délai de prise en charge avant le décès). Toutes les données collectées étaient reportées sur des fiches techniques pré établies et testées, saisies et analysées grâce aux logiciels CS pro 6.2 et SPSS 20. Les tests statistiques de comparaison utilisés étaient: le test du Khi 2 ou de Fischer en fonction des effectifs. Le seuil de significativité était retenu pour P < 0.05. Considérations éthiques: une clearance éthique était obtenue du comité d''éthique de l'Université de Yaoundé I et les autorisations de mener l'étude obtenue des administrations de chaque formation hospitalière impliquée. L'accès aux données était limité aux membres de l'équipe de recherche et ces données n'étaient destinées qu'à la recherche.

## Résultats

**Mortalité maternelle et état des patientes à l'admission dans les trois hôpitaux en 05 ans**: Les taux de décès maternels et l'état des patientes à l'arrivée dans les 3 structures sont représentés dans le Tableau 1.

**Tableau 1:** Mortalité maternelle et état des patientes à leur admission

Hôpitaux	Dossiers Retrouvés	Dossiersmanquants	EffectifTotal	NaissancesVivantes	Taux de MM/100000
Total	N=414 (%)	N =110 (%)	N=524 (%)	34 116(%)	1538.9
HGOPY	103 (24.9)	43 (39.1)	146 (27.9)	13 468 (39.5)	1 084.05
CHU	69 (16.7)	49 (44.5)	118 (22.5)	6 437 (18.9)	1 833.15
HCY	242 (58.4)	18 (16.4)	260 (49.6)	14 211 (41.6)	1 829.57
Etat des patientes référées à leur admission					
**Etat a l’admission**	Non référées N=94 (22.7%)	Référées N=320 (77.3%)	TotalN=414(100)		
Vivantes	92 (97.9%)	267 (83.4%)	359 (86.7)		
Décédées	2 (2.1%)	53 (16.6%)	55 (13.3)		

Pour un total de 31116 naissances vivantes (NV), le nombre de décès maternels était de 524, soit un taux de mortalité maternelle (TMM) de 1538.9/100 000 NV.

Sur un total de 414 dossiers retrouvés, 77.3 % étaient référées, parmi lesquelles 16.6% étaient arrivées décédées

**Etiologie des décès maternels**: Apres exploitation des 414 dossiers retrouves, nous avons ressorti la place occupée par chaque étiologie dans le Tableau 2.

**Tableau 2:** Etiologies des décès maternels

Etiologies	N=414	n (%)
Hémorragie du post-partum	110	2.6
Avortements	100	24.2
Hypertension artérielle	61	14.7
Hémorragie antépartum	44	10.6
Infection	39	9.4
GEUR	24	5.8
Autres	36	87
Total	414	100.0

L’hémorragie du post partum (HPP) était l’étiologie la plus fréquente (26.6 %). Les avortements et les GEU représentaient la 2^e^ et la 6^e^ étiologie avec respectivement 24.2 % (n=100) et 5.8% (n=24), soit une contribution de 30% (n=124) dans la mortalité maternelle

**Types d'avortements**: Parmi les 100 avortements, 60 (soit 60%) étaient provoqués et 40 (soit 40%) spontanés.

**Profil socio démographique des patientes décédées d'avortements ou de GEU**: Sur un total de 124 patientes, l'âge moyen était de 27.58 +/-6 ans, avec des extrêmes allant de 18 ans a 48 ans. Les autres caractéristiques sociodémographiques sont représentées dans le Tableau 3.

**Tableau 3:** Répartition des patientes décédées d’avortements ou de GEU selon les tranches d’âge, le statut matrimonial, le niveau d’instruction et la profession et les causes du décès

Cause des décès							
	GEUR		A provoqués		A spontanés		Total
Tranches d'âge							
	N=24(%)	P	N=60(%)	p	N=40(%)	P	N=124 (%)
[15-20]	2 (8.3)	1.00	5 (8.3)	0.888	2 (5)	0.888	9 (7.25)
[20-25]	9 (37.5)	0.017	21 (35)	0.03	11 (27.5)	0.898	41 (33.10)
[25-30]	6 (25.0)	0.180	13 (21.7)	0.747	11 (27.5)	0.024	30 (24.19)
[30-35]	4 (16.7)	0.784	14 (23.3)	0.861	8 (20)	0.861	26 (20.96)
[35-40]	3 (12.5)	0.059	4 (6.7)	0.074	5 (12.5)	0.074	12 (9.67)
>=40	0 (0)		3 (5)	0.908	3 (7.5)	0.908	6 (4.83)
**Statut matrimonial**							
	N=22 (%)	P	N=59 (%)	P	N=40 (%)	P	N=121(%)
Marié	6 (27.3)	0.228	10 (16.95)	0.32	14 (35)		30 (25)
Célibataire	16 (72.7)	0.368	49 (83.05)	0.001	26 (65)	0.08	91 (75)
**Niveau d’instruction**							
	N=	P	N=	P	N=	P	N=(%)
Analphabètes	0 (0)		0 (0)		3 (7.5)		3 (1.7)
Primaire	4 (18.2)	0.025	13	0.039	7 (17.5)	0.055	24(20)
Secondaire	15 (68.2)	0.23	36	0.006	24 (60.0)	0.153	75(62.5)
universitaire	3 (13.6)	0.018	10 (16.94)	0.010	6 (15.0)	0.921	19(15.8)
**Profession des patientes décédées**							
	**N=22(%)**	**P**	**N=59(%)**	**P**	**N=39(%)**	**P**	**N=120 (%)**
Salariée	1 (4.5)	0.969	2 (3.4)	0.969	2 (5.1)	0.001	5(4.2)
Ménagère	9 (40)	0.425	16 (27.1)	0.198	19 (48.7)	0.065	44(36.7)
Etudiant/Elève	6 (27.3)	0.138	21 (35.6)	0.007	9 (23.1)	0.508	36(30)
Commerçante	1 (4.5)	0.015	5 (8.5)	0.015	4 (10.3)	0.930	10(8.3)
Profession libérale	5 (22.7)	0.002	14 (23.7	0.048	3 (7.7)	0.548	22(18.3)
autres	0(0)		1(1.7)	0.013	2(5.1)	0.013	3(2.5)

La tranche d'âge de 20 à 24 ans était la plus représentée et était plus affectée par la GEU et les avortements provoqués. Cette tranche d’âge était suivie par celles de 25 à 29 ans et 30 à 34 ans.

Les célibataires étaient dominants à 75 % et mouraient plus (P=0.001) en ce qui concerne les avortements provoqués. Le niveau d'instruction secondaire était les plus représentées à 62.5%.

Les ménagères étaient les plus impliquées dans les avortements spontanés et GEU, tandis que les étudiantes /élèves étaient plus affectées (P=0.07) par les avortements provoqués.

**Antécédents**

**Méthodes contraceptives utilisées**: Sur 124 patientes décédées des suites d'avortement ou de GEU, 59 (soit 47.58%) utilisaient une méthode contraceptive. Les différentes méthodes contraceptives utilisées sont présentées dans le Tableau 4.

**Tableau 4:** Les méthodes contraceptives utilisées par les patientes décédées d’avortements ou de GEU

Méthodes contraceptives	N=59	Pourcentages (%)
Préservatifs	45	76.3
DIU	1	1.7
Pilule	2	3.4
Naturelle	11	18.6

Sur les 59 patientes, 45 soit 76.3% utilisaient le préservatif masculin. Une seule patiente soit 1.7% utilisait le dispositif intra utérin

**Statut sérologique au VIH des patientes décédées d'avortements et de GEU**: Sur les 124 patientes, 103 (83.1%) étaient séronégatives et 21 (16.9%) séropositives au VIH.

**Profil obstétrical des patientes décédées d'avortements ou de GEU**: La répartition de notre échantillon selon la parité, l'âge gestationnel et le nombre de consultations prénatales (CPN) est représentée dans le Tableau 5.

**Tableau 5:** Profil obstétrical des patientes décédées d’avortements ou de GEU

	GEUR		A. provoqués		A. spontanés		Total
Gravidité							
	N=22 (%)	P	N=58 (%)	P	N=39 (%)	P	N=119 (%)
Primigeste	3 (13.7)	0.98	8 (13.8)	0.230	8(20.6)	0.122	19(16)
Multigeste	17 (77.3)	0.232	35(60.4)	0.015	21(53.8)	0.138	73(61.3)
Grande multigeste	2 (9)	0.253	15(25.9)	0.281	10(25.6)	0.000	27(22.7)
**Age gestationnel (semaines de grossesse)**							
	**N=24 (%)**		**N=59 (%)**		**N=39 (%)**		**N=122 (%)**
< 8	10(41.7)	0.981	2(3.4)	0.322	2(5.1)	0.893	14 (11.5)
8- <15	14(58.3)	0.313	29(49.1)	0.296	1(2.6)	0.810	44(36)
15-28	0(0)		28(47.5)	0.0001	36(92.3)	0.0001	64(52.5)
**Consultations prénatales**							
	**N=24 (%)**		**N=60 (%)**		**N=40 (%)**		**N=124 (%)**
4 CPN et plus	0(0)		0(0)		3(7.5)		3(2.4)
Moins de 4 CPN	2(8.3)	0.306	3(5)	0.062	25(62.5)	0.062	30(24.2)
Aucune faite	22(91.7)	0.018	57(95)	0.001	12(30)	<0.0001	91(73.4)

Les grandes multi gestes (5 grossesses et plus) étaient exposées autant dans les avortements spontanés que les avortements provoqués, tandis que les multi gestes (1 à 4 grossesses) étaient plus impliquées dans les avortements provoqués. Par ailleurs, les avortements tardifs prédisposaient plus au décès que les avortements précoces. Les patientes n’ayant fait aucune CPN décédaient plus que les autres groupes (91 sur 124, soit 73.4% et P < 0.0001)

**Type et délai de prise en charge des patientes avant leur décès des suites d'avortements ou de GEU**: Le délai et le type de prise en charge des patientes avant leur décès des suites d'avortements ou de GEU sont représentés dans le Tableau 6.

**Tableau 6:** Délai et type de prise en charge avant le décès

	GEU		provoqué		spontanée		Total
Délai de prise en charge							
	N=23(%)	p	N=60 (%)	p	N=40 (%)	P	N=115 (%)
≤1 heure	8(4.10)	0.226	8 (14.0)	0.226	3(7.7)	0.302	19(16.5)
[1- 24 heures]	7 (36.80)	0.168	26(45.6)	0.002	15 (38.5)	0.662	48 (41.7)
[1 à 7Jours]	4 (21.10)	0.115	17(29.8)	0.257	19 (48.6)	0.005	40(34.8)
[1-4 semaines]	0(0)		4(7.1)	0.007	2 (5.2)	0.935	6(5.2)
> à un mois	0(0)		2(3.5)		0 (0)		2(1.7)
**Type de prise en charge**							
	**N=20(%)**		**57(%)**		**N=38 (%)**		**T=115(%)**
médical	8		44		35		87
chirurgical	12 (60%)		13(24.3%)		3		28

Sur un total de 115 patientes, 41.7% étaient pris en charge dans un délai de 1 à 24h et 34.8% entre 1 à 7 jours. La prise en charge par laparotomie d'urgence a été effectuée dans 24.3 % soit 13 cas /57 parmi les décédées d'avortements provoqués et 60 % soit 12 cas/ 20 parmi les GEU

**Complications maternelles ayant conduit au décès**: Les complications des avortements et GEU ayant conduit au décès ont été recensées dans les dossiers et sont représentées dans le Tableau 7.

**Tableau 7:** Complications des avortements et GEU ayant conduit au décès maternels

Complications		GEUR		A. Provoqués		A. Spontanés	
	Hémorragiques	N=23(%)	P	N=60(%)	P	N=40(%)	P
	Choc hémorragique	23 (95.8)	0.0001	27(45)	0.384	12 (30)	0.756
	Lacération filière génitale	/		5(8.3)		/	
	Rétention débris placentaire	/		7(11.7)		/	
	Aucune	0 (0)		21 (35)		28(70)	
	**Maladies HTA**	**N=23(%)**		**N=60(%)**		**N=40(%)**	
	Pré éclampsie antépartum	/		/		2 (5)	
	Eclampsie antépartum	/		/		4 (10)	
	Aucune	23(100)		60(100)		34 (85)	<0.0001
	**Infectieuses**	**N=0(%)**		**N=45(%)**		**N=0 (%)**	
	**Endométrite**	/		4(8.9)	0.003	2(7.4)	
	ATO	/		0 (0)		1(3.7)	
	Pelvipéritonite	/		7(15.6)		0(0)	
	**Péritonite**	/		13(28.9)	<0,0001	1(3,7)	
	**Choc de septique**	/		19(42.2)	<0,0001	7(25.9)	
	Autres			2(4.4)		16(59,3)	
	Autres	N=1(%)		N=49(%)		N=36(%)	
	Perforation uterine	/		2 (4.1)		0 (0)	
	Syndrome de HELLP	/		0 (0)		1 (2.8)	
	**CIVD**	/		1(2)		4 (11.1)	0.035
	Autre	1(4.2)		46 (93.9)		31 (86.1)	

Les complications hémorragiques concernaient principalement les GEUR suivi des avortements provoqués, tandis que les maladies hypertensives étaient retrouvées uniquement dans les avortements spontanés. Les causes infectieuses n'intéressaient que les avortements.

## Discussion

**Facteurs limitants**: Nous avons été limités dans notre étude par le fait que plusieurs dossiers comportaient très peu de renseignements nécessaires pour remplir la fiche technique ou n'étaient même pas retrouvés (Tableau 1). Par conséquent chaque analyse n'était faite que sur le nombre de variables valides. C'est une caractéristique inhérente aux études rétrospectives qui peut être corrigée à l'avenir par une bonne tenue de dossiers et surtout la mise en place d'un bon service d'archivage au sein de chaque hôpital.

**Mortalité maternelle et état des patientes à l'admission dans les trois hôpitaux en 05 ans**: Notre TMM de 1538.9/100000 NV (Tableau 1) est supérieur à celui de 782/100000 NV retrouvé au Cameroun lors des 4^e^ EDS-MICS 2011 [3], à celui de 1266.3/100 000 NV rapporté par Tebeu et coll. à l´hôpital régional de Maroua [10] et au TMM sans intervention en Afrique [11, 12]. Notre étude était réalisée dans les hôpitaux de références ou sont transférés les cas les plus graves. En effet 77.3% de patientes décédées étaient référées (Tableau 1) parmi lesquels 16.6% arrivaient déjà décédées.

**Etiologie des décès maternels** : L'hémorragie du post partum, les avortements et maladies hypertensives étaient les étiologies de mortalité maternelle les plus retrouvées soit 26.6%, 24.2% et 14.7% respectivement dans notre étude (Tableau 2). Les GEU rompues représentaient 58% (Tableau 2). Plusieurs auteurs ont observé que l'hémorragie était la première cause de mortalité maternelle [13]. Le taux de décès maternels de 24.2% (Tableau 2) lié aux avortements dans notre étude est largement supérieur à celui rapporté par l'OMS qui était de 13% [4, 5]. Il se rapproche de 23% retrouvé à Libreville [10]. Ceci peut se justifié par le fait que ces avortements soient souvent réalisés clandestinement et par des personnels non compétents et à cause de la loi restrictive sur les avortements dans notre pays. Notre taux décès maternels de 5.8% lies aux GEU est inférieur à celui de Libreville qui était de 9.7% [14]. Il se rapproche de 4% retrouvé en Suéde [12].

**Profil socio démographique des patientes décédées d'avortements et/ou de GEU**: L'âge moyen de 27.58 +/-6ans retrouvé dans notre étude (Tableau 3) est inférieur à l'âge moyen des mères qui décèdent en France qui est de 33.7 ans dans l'étude de Bouvier-Colle [15]. Cet âge correspond à la période de la pleine fécondité, surtout que l'âge de la primiparité est de 26 ans au Cameroun selon une étude réalisée par Tebeu et al au CHU [16]. Les tranches d'âge de 20 à 24 ans et de 25 à 29 ans (Tableau 3), les plus atteintes dans notre étude ont été retrouvées par d'autres auteurs au Ghana [17]. Les célibataires représentaient 75% dans notre étude, les élèves du secondaire 62.5%. Ces groupes sont vulnérables, du fait que les rapports sexuels sont souvent occasionnels, la contraception est rarement utilisée, les connaissances insuffisantes. Par conséquent leur capacité à se protéger est limitée, ce qui les expose à un risque plus élevé face aux grossesses non désirées [18], aux avortements et aux infections sexuellement transmissibles dont le VIH/Sida. Selon d'autres auteurs, ces groupes sont plus représentés parmi les patientes décédées d'avortements clandestins puisque c'est un moyen pour elles d'éviter la stigmatisation [19, 20].

**Antécédents**: Seulement 59 patientes sur 124 (soit 47.58%) utilisaient une méthode contraceptive (Tableau 4). Cette inaccessibilité à la planification familiale est commune à plusieurs pays d'Afrique, contre 4% en Europe [21]. Cette faible utilisation augmente les risques liés à la maternité et expliquent en partie les taux de mortalité observés. Or il est avéré qu'en agissant sur la prévalence contraceptive à travers un programme cohérent de planification familiale (PF) on peut réduire d'au moins 30% la mortalité maternelle [22]. La sérologie VIH positive était retrouvée chez 21 décédées d'avortements spontanés contre et 04 et 01 pour les décédés d'avortements provoqués et GEUR respectivement.

**Profile obstétrical**: Les multigestes étaient les plus représentées (36.1%) (Tableau 6). Ceci pourrait s'expliquer par les besoins non satisfaits en planification familiale, qui exposent ces dernières aux grossesses non désirées [18] et donc aux avortements clandestins. Cet argument ressort dans l'étude de Sone et coll. en 2015 qui retrouve 5.1% de cause d'avortement due au fait d'avoir un jeune bébé en même temps qu'est survenue la grossesse non désirée [23]. Les avortements survenus entre la 15^ème^ à la 28^ème^ semaine de grossesse étaient significativement à risque de décès maternel comparé à ceux survenant avant la 8^ème^ semaine de grossesse et ceux compris entre 8 et 14 semaines de grossesse. Ceci corrobore avec les résultats de Bartllet aux USA en 2004 qui a trouvé que les avortements survenus entre la 16 à la 20 semaine étaient relativement lies au décès maternels liés aux avortements comparés à ceux survenant avant la 8 et entre la 13 à la 15 semaine de grossesse [24]. Environ 2.4% des décédés avaient effectués au moins 4 CPN et 73.4% qui n'avaient effectué aucune (P=0.000) (Tableau 6). L'absence de CPN effectuée et la réalisation de CPN de mauvaise qualité s'est révélé être un facteur de risque de décès maternel dans notre étude avec un P= 0.000 pour les avortements provoqués et un P=0.001 pour les avortements spontanés. Seulement 1.2% d'entre elles ont effectué 4CPN comme recommandées par l'OMS [22] avant l'année 2017. La prise charge chirurgicale par laparotomie d'urgence a intéressée 24.3% pour les GEU (Tableau 6). Le délai de prise en charge avant le décès des patientes dans notre série était estimé à plus d'une heure et moins de 24 heures chez 41.7%. Ceci s'expliquerait par les trois retards [25]: le retard de prise de décision d'aller à l'hôpital ou de référence vers les hôpitaux de niveau compétent, le retard d'accéder à l'hôpital et le retard de prise en charge dans la formation sanitaire à cause du personnel non compétent. Par ailleurs le problème socioéconomique (le coût financier) et/ou du plateau technique limité à l'instar des dérivés sanguins dans nos hôpitaux, ne sont pas à négligés dans la contribution de la mortalité maternelle. Concernant les complications (Tableau 7): les complications hémorragiques dominaient à 50%, ce résultat est supérieur comparé à celui de Srinil en Thailand en 2011 [26]; et pourrait s'expliquer par la différence de plateau technique entre ces deux pays et au problème de déficit en dérivés sanguins dans nos hôpitaux. Les complications infectieuses suivaient avec un taux à 36.1% de choc septique et 19.4% de péritonites; ces taux sont en accord avec certains auteurs qui ont retrouvés que la mortalité due au sepsis post abortum chez leurs patients était élevée et compris entre 20% à 50% [27].

## Conclusion

Les avortements et grossesse extra-utérines occupent la 2^ème^ et la 6^ème^ place dans la mortalité maternelle au cours de ces cinq années, avec des prévalences de 24.2% et 5.8% respectivement, soit une contribution cumulée de 30%. Les complications hémorragiques et infectieuses étaient prédominantes. L'intensification de la planification familiale permettrait de prévenir les grossesses non désirées et IST au sein des populations. Par ailleurs un recyclage et/ou formation du personnel médical et paramédical dans le counseling s'avèrent nécessaires pour faciliter l'acceptation de cette situation par des couple en cas de grossesses non désirées. La promotion d'une politique nationale pour la transfusion sanguine et la création d'un centre national de production et fabrication de dérivées sanguins permettraient d'éviter les causes hémorragiques des décès par avortement et grossesses extra utérine.

### Etat des connaissances actuelle sur le sujet

- L'avortement contribue pour 13% de décès maternels dans le monde (4.5) et 30 à 40% en Afrique;
- La GEU est responsable 10% de décès maternels au premier trimestre de grossesse.

### Contribution de notre étude à la connaissance

- Les avortements et les grossesses extra-utérines ont contribué respectivement pour 24.2% et 5.8% dans la mortalité maternelle dans les hôpitaux de référence de Yaoundé soit une contribution cumulée de 30% dans la mortalité maternelle au cours de ces cinq années;
- Le profil épidémiologique de la patiente qui décède est: âge de 20 à 30 ans, célibataires, de niveau d'instruction secondaire pour la majorité, primigestes pour celles décédées d'avortement provoqués et grande multi gestes pour celles décédées d'avortement spontané.

## Conflits d'intérêts

Les auteurs ne déclarent aucun conflit d'intérêts.

## Remerciements

Nous remercions les administrations et le personnel des hôpitaux (HGY, HCY et HGOPY) qui nous ont autorisé et facilité la réalisation de cette étude.
